# Imperforate tracheary elements and vessels alleviate xylem tension under severe dehydration: insights from water release curves for excised twigs of three tree species

**DOI:** 10.1002/ajb2.1518

**Published:** 2020-08-11

**Authors:** Kenichi Yazaki, Delphis F. Levia, Akiko Takenouchi, Makoto Watanabe, Daisuke Kabeya, Naoko H. Miki, Haruhiko Taneda, Mayumi Y. Ogasa, Michio Oguro, Shin‐Taro Saiki, Hiroyuki Tobita, Kenji Fukuda

**Affiliations:** ^1^ Department of Plant Ecology Forestry and Forest Products Research Institute (FFPRI) Tsukuba Ibaraki 305‐8687 Japan; ^2^ Departments of Geography & Spatial Sciences and Plant & Soil Sciences University of Delaware Newark DE 19716 USA; ^3^ Research Center for Structural Materials National Institute of Materials Science (NIMS) Tsukuba Ibaraki 305‐0047 Japan; ^4^ Graduate School of Environmental and Life Science Okayama University Okayama 700‐8530 Japan; ^5^ Graduate School of Science The University of Tokyo Tokyo 113‐0033 Japan; ^6^ Kansai Research Center Forestry and Forest Products Research Institute (FFPRI) Kyoto Kyoto 612‐0855 Japan; ^7^ Department of Forest Vegetation Forestry and Forest Products Research Institute (FFPRI) Tsukuba Ibaraki 305‐8687 Japan; ^8^ Graduate School of Agricultural and Life Sciences The University of Tokyo Tokyo 113‐8657 Japan

**Keywords:** *Abies*, capacitance, *Cercidiphyllum*, cryo‐SEM, micro focus x‐ray CT, *Quercus*, water storage, xylem structure

## Abstract

**Premise:**

Water stored in the xylem of woody plants is important for supporting the transpiration stream under prolonged drought, yet the source of stored water within the xylem during drought remains unclear. Insights into xylem water utilization during drought will uncover the adaptation strategies of the test species to stress.

**Methods:**

To fill the existing knowledge gap, we excised twigs of *Abies firma* (Japanese fir, conifer), *Cercidiphyllum japonicum* (katsura tree, diffuse‐porous) and *Quercus serrata* (konara oak, ring‐porous) to quantify interspecific variation of water transfer in xylem corresponding with increasing cumulative water release (CWR) using micro x‐ray computed tomography and cryo‐SEM.

**Results:**

For all species studied, the main components of water storage within the operating range of water potential were not living cells but cavitation release and capillaries. *Abies firma* maintained water in the earlywood‐like cells, for possible maintenance of the transpiration stream. *Cercidiphyllum japonicum* maintained water in its vessels over 200 kg m^‐3^ of CWR, while *Q. serrata* lost most of its water in vessels with increasing CWR up to 100 kg m^‐3^. *Cercidiphyllum japonicum* exhibited a higher water storage capacity than *Q. serrata*. Under high CWR, narrow conduits stored xylem water in *C. japonicum* and imperforate tracheary elements in *Q. serrata*.

**Conclusions:**

Among the species examined, increasing CWR appears to indicate differential utilization of stored water in relation to variation of xylem structure, thereby providing insight into the interspecific responses of tree species to drought.

Woody species have evolved various defense mechanisms against hydraulic dysfunction in xylem conduits under prolonged water deficit, such as initiating foliar shedding or osmotic adjustment to avoid or mitigate dehydration stresses. The capacity of water storage in xylem has been reported to determine the hydraulic conductance and daily water potential of leaves (Meinzer et al., 2003, 2009). A trade‐off between water storage and mechanical strength in xylem also has been reported (Jupa et al., 2016; Pratt and Jacobsen, 2016), and it will determine the water‐use strategy of the species under fluctuating water conditions. Given the increasing uncertainty of water supply to trees under climate change, water stored in xylem is an important topic worthy of detailed examination to facilitate a better understanding of how long a particular species can maintain water potential for the avoidance of hydraulic failure (Tyree and Yang, 1990; Meinzer et al., 2003, 2009; Richards et al., 2014; Jupa et al., 2016; Pratt and Jacobsen, 2016; Knipfer et al., 2017, 2019).

The amount of water stored in xylem can be estimated from the relationship between the amount of water released and xylem water potential (Tyree and Yang, 1990; Meinzer et al., 2003). Tyree and Yang (1990) suggested the three mechanisms by which water stored within saturated xylem could be released to combat dehydration: (1) capillary storage—water is stored mainly in the lumen of dead fibers and tracheids (i.e., imperforate tracheary elements [ITEs]: unicellular tracheary elements that lack a perforation plate), in the tapered end of cavitated conduits, or in intercellular spaces; (2) elastic storage—water is located in the symplast of living cells such as parenchyma cells in bark and/or xylem; and (3) cavitation release—water comes from functional conduits. It has been widely accepted that the water released from capillary storage at the initial decline of water capacitance can facilitate the maintenance of water potential, while the water stored from cavitation release may alleviate drought stress and recovery (Tyree and Yang, 1990; Pratt et al., 2007; Richards et al., 2014; Knipfer et al., 2017). Tyree and Yang (1990) further suggested that elastic cells, such as parenchyma, are important for delayed water release from xylem osmotically. In contrast, Jupa et al. (2016) reported positive relationships between the relative amount of cellular space of vessels and/or ITEs and the amount of water storage. Knipfer et al. (2019) focused on the water dynamics between vessels and ITEs in the xylem of intact juvenile trees under prolonged dehydration and rehydration using micro focus x‐ray computed tomography (µCT). Whereas the importance of the role of ITEs to water storage in tree has been raised, the precise locus of water storage and dynamics in the xylem cells under progressive dehydration remains unclear (Jupa et al., 2016; Knipfer et al., 2019).

Water storage capacity in the xylem has long been known to depend on the density and anatomical structure of the wood (Bucci et al., 2004; Pratt et al., 2007; Meinzer et al., 2008; Jupa et al., 2016). The number of ITEs is especially relevant since they are a major determinant of wood density for a species (Fortunel et al., 2014). Some xylem cells are classified as ITEs that contain vasicentric tracheids, fiber tracheids, or libriform fibers in angiosperm species; except for some living ITEs, almost all ITEs consist of dead cells without protoplasm (Wheeler et al., 1989; Ohtani, 2000; Sano et al., 2011). One of the major roles of ITEs is to support the woody frame of the tree. Another role of ITEs is the support of water transfer of vessels (Sano et al., 2011; Pratt et al., 2015; Barotto et al., 2016). However, the role of water storage in these ITEs under dehydration is still unclear, although the structure involved with higher wood density is regarded as an adaptive trait to enduring water shortages.

To gain a more complete understanding of water storage in xylem, we need to clarify how each cell holds water as the cell progresses toward dehydration. The amount of water storage within xylem is estimated as “capacitance”, defined as the amount of water released from xylem per gradient of water potential (Tyree and Yang, 1990; Meinzer et al., 2003). However, relatively little work has focused on water dynamics in xylem tissue as stored water becomes depleted (Knipfer et al., 2019). Cryo‐scanning electron microscopy (cryo‐SEM) allows high‐resolution observations of water distribution in xylem cell (e.g., Yazaki et al., 2019), but it has seldom been used to investigate water dynamics in ITEs (Sano et al., 2011). Recently, several nondestructive imaging techniques, such as µCT or MRI, have helped clarify water dynamics in intact woody species when water levels fluctuate (Cochard et al., 2015; Fukuda et al., 2015; Choat et al., 2016; Knipfer et al., 2017, 2019; Ogasa et al., 2019). While these studies have shown the location of vulnerable xylem under dehydration, or refilling of empty conduits with irrigation following dehydration in intact plants, there is still a knowledge gap with regard to interspecific differences in the water dynamics of xylem tissue in relation to decreasing xylem capacitance.

In this study, we sought to clarify the role of water storage within xylem tissues by examining the water dynamics of xylem segments in twigs in relation to the changes in capacitance using direct observation of water in each cell. We especially focused on the water behavior in xylem cells under severe dehydration that should influence the mortality of the species (Barigah et al., 2013; Urli et al., 2013; Hammond et al., 2019). We further compared the differences in xylem water dynamics under a progression to dehydration among three species, which have different xylem structures. This comparison should yield a better understanding of xylem capacitance under dehydration stress across a range of xylem types. We employed cryo‐SEM and commercial desktop µCT for low‐cost frequent observations. By comparing three tree species, we sought to bridge cellular, plant, and environmental scales by clarifying the availability of stored water in xylem under prolonged drought. Knowledge of the differential responses of these species to a reduction in xylem capacitance will better inform our theories of the drought tolerance of trees associated with wood density, thereby advancing our knowledge of tree–water interactions.

## MATERIALS AND METHODS

### Sample harvesting

**Figure 1:**
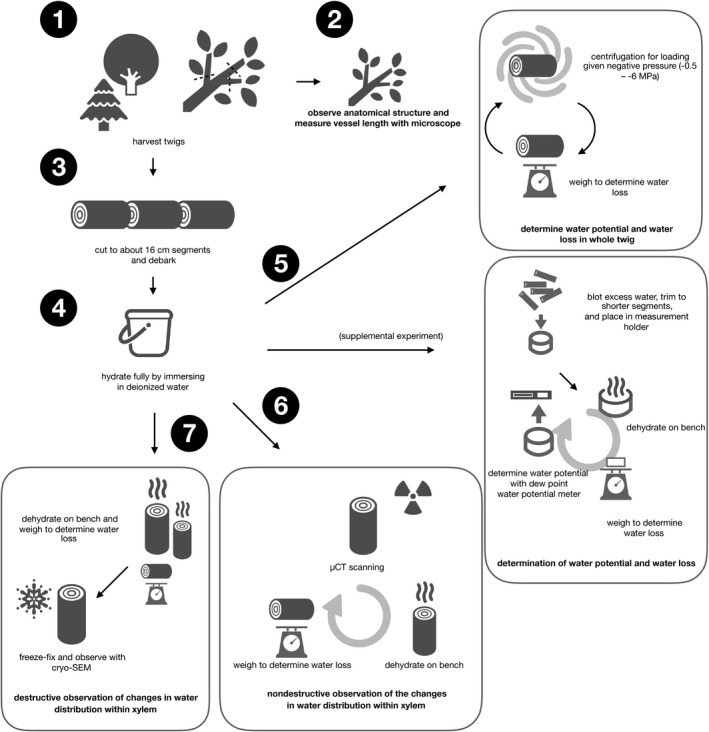
Schematic of protocol to measure capacitance and observe anatomical structure or water status used in this study.

We sampled twigs from mature trees of *Abies firma* Siebold et Zucc. (Japanese fir, Pinaceae, conifer), *Cercidiphyllum japonicum* Siebold et Zucc. ex Hoffm. et Schult. (katsura tree, Cercidiphyllaceae, diffuse‐porous) and *Quercus serrata* Thunb. (konara oak, Fagaceae, ring‐porous) grown in the tree garden of the Forestry and Forest Products Research Institute (Tsukuba, Japan, 36°00′N, 140°08′E, 20 m a.s.l.). Several branches greater than 5 years old and approximately 2 cm in diameter were cut from one to three individuals of each species. From these older branches, more than 40 1‐ to 2‐year‐old twigs of approximately 16 cm long, which is more than three times as long as the vessel length of each angiosperm (see next section for more detail), were harvested for each species. Bark was removed from the segments immediately. Then, all twigs were immersed in deionized water and vacuumed overnight to saturate them with water to estimate the maximum capacity of water storage in the xylem. A schematic depicting the sequence of tasks is shown in Fig. 1. It is important to note that this study does not require the relaxation of xylem tension during harvesting twigs to avoid tension‐induced cavitation (i.e., cutting artifact; Wheeler et al., 2013; Ogasa et al., 2019; Yazaki et al., 2019) because its objective is to explore the nature of water dynamics from the hydrated xylem cells by immersion.

### Anatomical measurements of two angiosperms

Within the transverse sections, we estimated the relative area of parenchyma cells, vessels, and ITEs and mean vessel length in segments of two angiosperms using optical microscopy. Four 1‐ to 2‐year‐old twigs for each angiosperm were sampled and cut into smaller pieces. The pieces were fixed with 50% ethanol and transverse sections of 12–15 µm thick were cut with a sliding microtome from the basal end of the fixed segments. Sections were stained with 1% (v/v) aqueous safranin and mounted in Bioleit (Okenshoji Co., Tokyo, Japan). Digital images (1372 × 1024 pixels in each image, 1.85 pixel/µm) were acquired (Appendix S1) and joined from two radial directions of the transverse section using a 10× objective lens. Image J software (U. S. National Institutes of Health, Bethesda, MD, USA, https://imagej.nih.gov/ij/) was used for all image analyses. Axial and radial parenchyma cells were distinguished based on their contents and selected manually using a graphics tablet. Whole vessel lumina were selected and total area of vessel lumen was measured automatically using the function Analyze particles in Image J software. Each fraction of the transverse area of a parenchyma cell or the total vessel lumen was estimated by dividing total transverse area of a parenchyma cell or total vessel lumen by the area of the region of interest (ROI) in the image. The fraction of ITEs plus cell walls was calculated by subtracting the sum of the fraction of parenchyma and vessel lumen from the area of the ROI. The values from two directions in each section were averaged.

Vessel length was estimated using the silicone–injection method (Hacke et al., 2007). In brief, we sampled three other twigs about 20 cm long from each of the two species and fully flashed them. Silicone (RTV‐141, Rhodia, Cranbury, NJ, USA) mixed with a drop of 1% (w/w) fluorescent brightener (Uvitex OB, Sigma‐Aldrich Japan, Tokyo, Japan) in chloroform per gram of silicone was injected into the twig at about 150 kPa overnight. After silicone curing, we cut a 15 µm thick transverse section at 0.5 cm, 1.0 cm, 2.0 cm, 4.0 cm, 8.0 cm, and 16 cm from the injected end of twigs. Each section was observed using fluorescence microscopy (330 to 380 nm excitation, 420 nm emission, 80iRT equipped with UV‐2A, Nikon, Tokyo, Japan). The number of vessels with injected silicone was counted in each image acquired from fluorescence observation using Image J software. The distribution of vessel lengths was calculated using a macro program in the spreadsheet file downloaded from the section on vessel lengths on the methods and computer programs web page of the John Sperry Laboratory, University of Utah (http://sperry.biology.utah.edu/methods.html#vessel_lengths).

Significant differences for each variable between two species were evaluated using Student’s *t*‐test.

### Water release curve in xylem

The relationships between the amount of water release and xylem water potential and xylem capacitance (*C*) of each species were determined for a separate set of twigs using two methods (Fig. 1). The first involved the centrifugation of whole segments of twigs, which were brought to a given water potential (Domec and Gartner, 2002; Cochard et al., 2010, 2015). Specifically, the immersed twig was trimmed under water at both ends with a sharp razor blade and resized to 14.5 cm, which is the maximum size for our centrifuge system (Model 3700 equipped with a custom rotor, Kubota, Tokyo, Japan). Any excess water on the surface of the segment was wiped off with a paper towel. The sides of the segment were tightly wrapped with parafilm to prevent excessive water loss. The segment was attached to the rotor with water‐filled reservoirs to immerse both ends of the segment in water during centrifugation, then centrifuged for 2 min to attain the target water potential (Alder et al., 1997). The segment was detached from the rotor and weighed with an electric balance after centrifuging. This centrifuging and weighing procedure was repeated stepwise from –0.5 to approximately –6 MPa, the limitation of our system due to the rotor size.

The second method sought to determine the water potential of excised short segments of the sample psychrometrically (Meinzer et al., 2003; Richards et al., 2014; Jupa et al., 2016). Any excess water on the surface of immersed twigs was wiped off with a paper towel. Then, the twigs were cut and divided into shorter subsamples, approximately 1 cm in axial length and 2–3 mm in width, with a sharp blade. Approximately 30 subsamples were put into a 15‐mL sample holder of stainless steel and spread over the bottom of the holder. The sample holder was weighed with an electric balance. Xylem water potential of the twig subsamples (*ψ*_x_) was determined with a dew point potential meter (WP4‐T, METER Group, Pullman, WA, USA) after stabilization of the water potential value of each measurement (at least 30 min) in the laboratory at 25°C and 45–50% relative humidity. After reweighing the sample holder, the twig subsamples in the sample holder were bench‐dried in the laboratory for 30–60 min. After each bench‐drying and weighing, *ψ*_x_ was determined. The bench‐drying and determination of *ψ*_x_ was repeated eight to nine times. After completion of the repeated measurements of *ψ*_x_, the twig subsamples were oven‐dried (70°C, 48 h), and the sum of the dry mass of the twig subsamples was computed. For each species, three sample sets were used to determine the relationship between *ψ*_x_ and mass.

Cumulative water release (CWR, kg m^–3^), defined as the cumulative amount of water loss per saturated wood volume, can be used to compare dehydration status among various sample sizes (Meinzer et al., 2003). We calculated CWR as:

(1) $$\text{CWR}=\left(W_{s}‐W\right)/\left(W_{d}/\text{BD}\right),$$

where *W*_s_ is the saturated mass of the subsampled twigs and *W* is the mean mass of the twigs before and after each measurement of xylem water potential. *W*_d_ is the sum of the dry mass of subsamples used for the psychrometer, or the dry mass of the samples used for the centrifugation, and BD is the basic density (kg m^–3^) of wood of the species, determined by water displacement methods using a separate set of 30–40 twigs per species. The difference in basic density among species was statistically evaluated by one‐way ANOVA. A hyperbolic function was applied to estimate the relationship between CWR and *ψ*_x_ (Meinzer et al., 2003; Jupa et al., 2016):

(2) $$\text{CWR}=a\left(‐\psi_{x}\right)/\left[b+\left(‐\psi_{x}\right)\right],$$

where *a* and *b* are species‐specific constants indicating maximum CWR and *ψ*_x_ at 50% CWR, respectively. In this study, we assumed *a* and *b* were also affected by individuals. A nonlinear mixed effects model was fitted with statistical software (R version 3.3.3, R Core Team, R Foundation for Statistical Computing, Vienna, Austria; http://www.R‐project.org) with the nlme package. The model included individual effects as random effects. Each independent model equation was fitted to the data set of each species. We selected the best estimator of *a*, *b*, and the efficacy of random effect on each model according to the value of Akaike’s jnformation criterion (AIC). Statistical differences for each parameter among species were evaluated by species‐specific parameters using dummy variables in the selected model.

Equation 2 in each species was differentiated with respect to *ψ*_x_ to determine *C*, defined as *C* = ΔCWR / Δ*ψ*_x_, as:

(3) $$C=△\text{CWR}/△\psi_{x}=ab/{\left[b+\left(‐\psi_{x}\right)\right]}^{2}.$$

### Nondestructive observation with desktop µCT

The water dynamics of the xylem of two or three saturated twigs of each species were observed nondestructively using a commercial µCT (SMT‐160CT, Shimadzu, Tokyo, Japan) corresponding with the variation of capacitances. To reduce blurring of CT images, we adjusted the length of each twig to approximately 12 cm for *C. japonicum* and *Q. serrata* and approximately 4 cm for *A. firma*. Each of the saturated subsampled twigs was tightly wrapped with parafilm to prevent dehydration during µCT scanning.

The distance from the x‐ray source to the object (SOD) was set to 12 mm and the distance from the x‐ray source to the image intensifier (SID) was set to 250 mm. The voltage of the x‐ray source was 44–47 keV. A full‐rotated cone scan was conducted to the axial center of each twig with 1200 views, with a slice pitch (length between slices) of 0.0145 mm, and a slice thickness of 0.0052 mm. A total of 512 images was obtained in the *z* direction, which have 512 × 512 pixels in the *xy* plane. The size of field of view in the *xy* plane was 2.62 × 2.62 mm (i.e., resolution of images was 5.23 µm/pixel). After each observation by µCT, one transverse side of the specimen was unwrapped to be bench‐dehydrated at room temperature and humidity (25–30°C, 30–40% RH) or dehydrated in controlled conditions (30–40°C, approximately 10%RH) in a portable incubator chamber (J. P. CULTURE III, AS ONE Co., Osaka, Japan), for 60–120 min. We repeated weighing and CT scanning several times until the dehydration progressed sufficiently. CT images were analyzed using Image J software. To assess the water dissipation in xylem, we compared the time series of the same transverse plane derived from the reconstructed 3D CT image. The dry mass of each specimen was measured after all CT scans. The CWR of the specimen was calculated at each CT scan from the decrease in mass during dehydration using Eq. 1.

We determined the percentage of cavitation area of tracheids (*A. firma*) or vessels (two angiosperms) in the transverse surface in each species at given CWR using sequential CT images and Image J software.

As a supplement, we also conducted similar µCT observation using short segments (approximately 4 cm long; Appendices S2 and S3).

### Cryo‐SEM observation to estimate water distribution in ITEs

We determined the distribution of water in each individual type of xylem cell under several CWRs for two angiosperm species via cryo‐SEM. The other saturated twig subsamples of *C. japonicum* and *Q. serrata* were wrapped and bench‐dried as for the observation of µCT described above. The subsample was weighed every 15–120 min to monitor the dehydration. After reaching several targets of CWR (0–500 kg m^‐3^ in *C. japonicum*, or 0–400 kg m^‐3^ in *Q. serrata*), each subsampled twig was freeze‐fixed by immersion in liquid nitrogen for a few minutes. The specimens from the freeze‐fixed samples were trimmed with a cryostat‐microtome (CryoStar NX70, Thermo Fischer Scientific K.K., Tokyo, Japan) to smooth the transverse surface. The specimens were observed with cryo‐SEM (JSM‐6510 attached cryo‐SEM unit; JEOL, Tokyo, Japan) under an accelerating voltage of 3 kV and cold stage temperature of approximately –130°C.

For the two angiosperms, cryo‐SEM images were used to determine the percentage of cavitation area of ITEs. The images of 350× (1280 × 960 pixels in each image, 3.5 pixel/µm), which allowed distinguishing the water status of cell lumen in ITEs, were acquired from the at least two directions of radial files of xylem cells. The cells neighboring the pith (primary xylem) were excluded from analysis. For *Q. serrata*, cavitated ITEs were distinguished between vasicentric tracheids and libriform fibers by their appearance; ITE cells with thin cell walls, surrounding vessels were regarded as vasicentric tracheids (Wheeler et al., 1989; Ohtani, 2000).

We also verified the distribution of water within the xylem of *A. firma* as determined from µCT images via cryo‐SEM. The other saturated twig subsamples of *A. firma* were bench‐dried to partially dehydrate their xylem. The dehydration time was approximately 1 h for *A. firma*. Each subsampled twig was scanned by µCT, after freeze fixation as described above. The freeze‐fixed specimens were trimmed at the transverse surface in the reconstructed 3D CT images with the cryostat‐microtome. Then, we compared the slice from the CT images corresponding to the respective cryo‐SEM image. Similar verification was conducted for *Q. serrata*, which has large conduits (Appendix S4).

## RESULTS

### Anatomical features and basic density

The anatomical features of the two angiosperms are summarized in Table 1. Vessel length was approximately half the length of the segments used for the dehydration experiments in this study; thus, we were able to assume that the open vessel artifact for dehydration was reduced. *Cercidiphyllium japonicum* had exclusively uniseriate rays and no axial parenchyma cells, while *Q. serrata* had larger rays with multiseriate and many apotracheral parenchyma cells. The two angiosperms, however, had a similar percentage of total parenchyma cells (Table 1). In contrast, *Q. serrata* had more ITEs but fewer vessels (i.e., sum of the lumen area) than in *C. japonicum* (Table 1). The basic density of xylem of all three species was significantly different (*p* < 0.01, ANOVA) with *Q. serrata* having the highest xylem density (Table 2).

**Table 1:** Vessel length and fraction of the transverse area made up by each cell type in the xylem of twigs of two angiosperms.

			Fraction of transverse area of each cell type (%)									
	Vessel length (m)		ITEs (+ cell wall)		Parenchyma (ray + axial)		Ray parenchyma		Axial parenchyma		Vessel (lumen)	
Species	Mean	SD	Mean	SD	Mean	SD	Mean	SD	Mean	SD	Mean	SD
*Cercidiphyllum japonicum*	0.037	0.011	68.2	3.4*	10.1	1.6	10.1	1.6	0.0	0.0*	21.7	1.8*
*Quercus serrata*	0.047	0.010	76.7	5.2	15.6	4.7	8.8	4.1	6.7	2.6	7.8	3.3

Notes

*p < 0.01, Student’s *t*‐test (df = 7, *n* = 3–4).

**Table 2:** Basic density of twig segments of the test tree species.

	Basic density (g m^‐3^)	
Species	Mean	SD (*n* = 36–42)
*Abies firma*	0.455	0.065
*Cercidiphylum japonicum*	0.532	0.045
*Quercus serrata*	0.648	0.055

Notes

One‐way ANOVA revealed significant effects of species (*F*_2, 65_= 173.6, *p* < 0.01).

### CT images corresponding to water release

**Figure 2:**
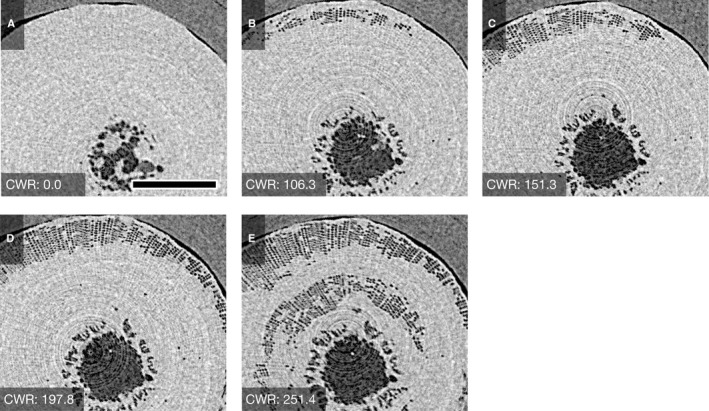
Typical sequential µCT images showing water distribution in the xylem of *Abies firma* (conifer) and water status of xylem during bench‐drying. Estimated values of cumulative water release (CWR) are shown in each panel. Scale bar = 200 µm.

The desktop µCT provided the temporal changes in the distribution of water within xylem tissues of the studied species (Figs. 2, 4, and 5). The segments observed via µCT were at least twice as long as the vessel lengths in each of the two angiosperms, indicating the negligible effects of open vessel artifacts on our results (Table 1).

**Figure 3:**
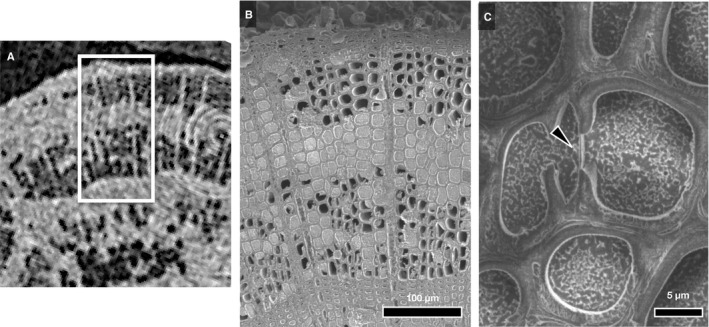
Cryo‐SEM images of water distribution within xylem of *Abies firma* after bench‐drying. Large tracheids in center of xylem were able to hold water with dehydration, while some narrower tracheids lost their water (A). Tracheids in the outer xylem exposed to air by the experimental removal of bark were able to hold water, indicating that dehydration did not progress radially. Pit aspiration was observed between the tracheids, which were filled with water (arrowhead) (B). Note that this figure was not the same sample observed by µCT in Fig. 2.

**Figure 4:**
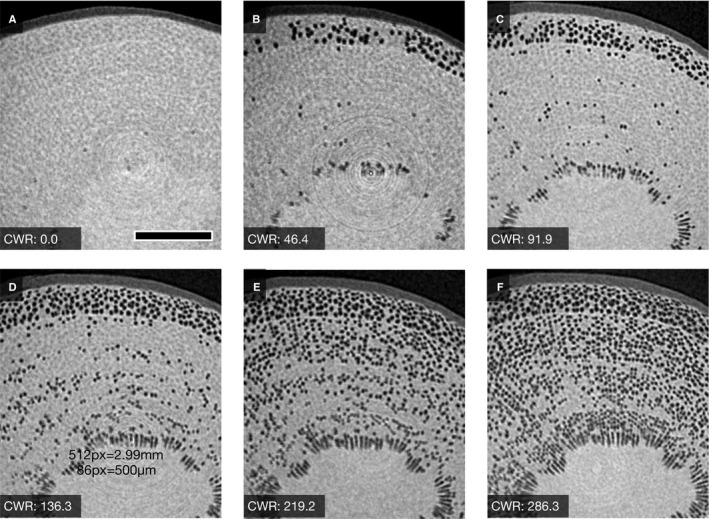
Typical sequential µCT images showing water distribution in the xylem of *Cercidiphyllum japonicum* (diffuse‐porous) and water status of xylem with bench‐drying. Estimated values of cumulative water release (CWR) are also shown in each panel. Scale bar = 200 µm.

In the xylem of *A. firma*, water dissipated in the latewood‐like tracheids in the outer growth ring at nearly 100 kg m^‐3^ of CWR following dissipation in the pith, and progressed from the outer xylem to the inner xylem (Fig. 4B, D). However, the tracheids located in intermediate portions of the xylem retained water at approximately 250 kg m^‐3^ of CWR as dehydration progressed (Fig. 4E). Cryo‐SEM images corresponding to the µCT image showed that many earlywood‐like tracheids retained water under the dehydrated condition (Fig. 3A, B). Some of the pit membranes in the bordered pits among tracheids filled with water were aspirated (Fig. 3C).

*Cercidiphyllium japonicum* lost its water in xylem from vessels located along the annual ring boundaries (Fig. 4B–D), followed by dissipation of water from vessels located in the outer xylem in each annual ring (Fig. 4E). Although almost all vessels seemed to have cavitated lower than 250 kg m^‐3^ of CWR (Fig. 4E), several vessels were able to hold water under higher dehydration status (Fig. 4F).

**Figure 5:**
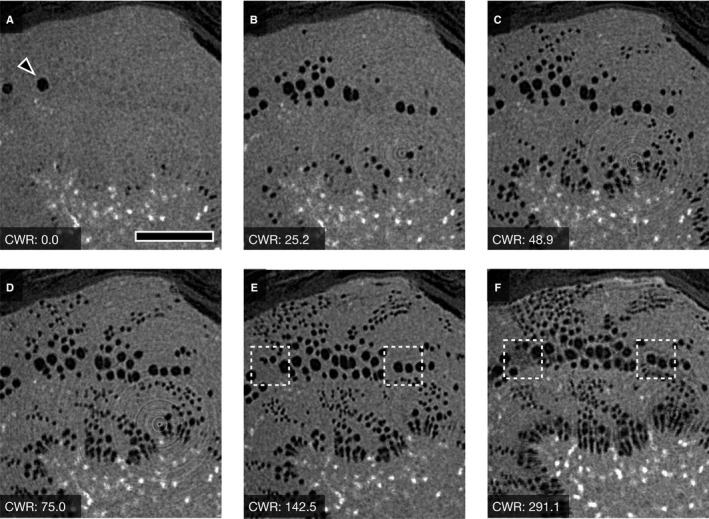
Typical sequential µCT images of the xylem of *Quercus serrata* (ring‐porous) during dehydration. The arrowhead in panel A indicates suspected artifact of cavitation because the vessel is open‐ended. Note that cavitation is shown in the wood fiber (the area surrounded by dotted squares) in panel F, as opposed to panel E. Scale bar = 200 µm.

Although the xylem of *Q. serrata* exhibited a few empty vessels at saturated conditions (Fig. 5A), these empty vessels were found less frequently (refer to Fig. 7). *Quercus serrata* first lost most of its water from the earlywood vessels located at the boundary of growth rings; smaller vessels lost water later (Fig. 5B–D). Almost all vessels lost water by 140 kg m^‐3^ of CWR (Fig. 5E). The xylem of *Q. serrata* seemed to release water in the ITEs, especially surrounding vessels under conditions of high dehydration and lower capacitance (Fig. 5E and F, dotted squares).

**Figure 6:**
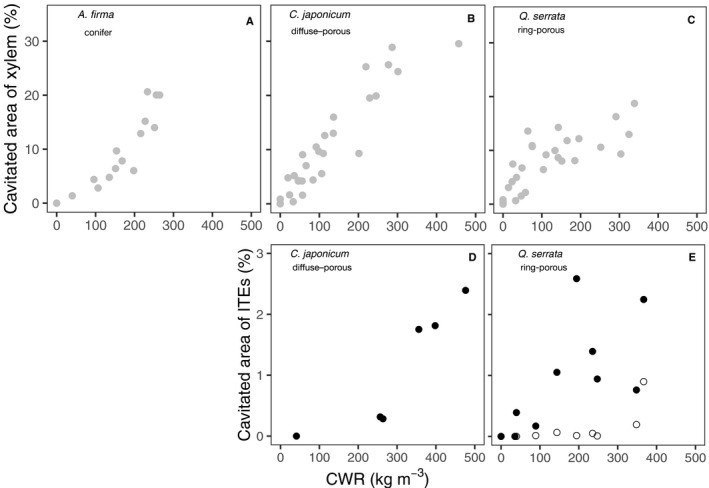
Percentage of cavitated area of xylem calculated from sequential µCT images of (A) *Abies firma*, (B) *Cercidiphyllum japonicum*, and (C) *Quercus serrata* and percentage of the cavitated area of ITEs measured in cryo‐SEM images of (D) *C. japonicum*, and (E) *Q. serrata*. The results of µCT images of two (*A. firma*) or three (*C. japonicum* and *Q. serrata*) individuals are merged. In panels D and E, closed circles (●) and open circles (○) denote the value of libriform fiber and vacisentric tracheid, respectively, and each symbol represents the mean value of several images for an individual.

The changes in cavitated area of xylem with the increase in CWR showed different patterns among the species (Fig. 6A–C). While cavitated area increased linearly within the range of changes in CWR of the segments for both *A. firma* and *C. japonicum*, the cavitated area reached a maximum of approximately 100 kg m^‐3^ of CWR in *Q. serrata,* indicating that almost all vessels should be empty.

### Water distribution in xylem cells of two angiosperm with increase in CWR

**Figure 7:**
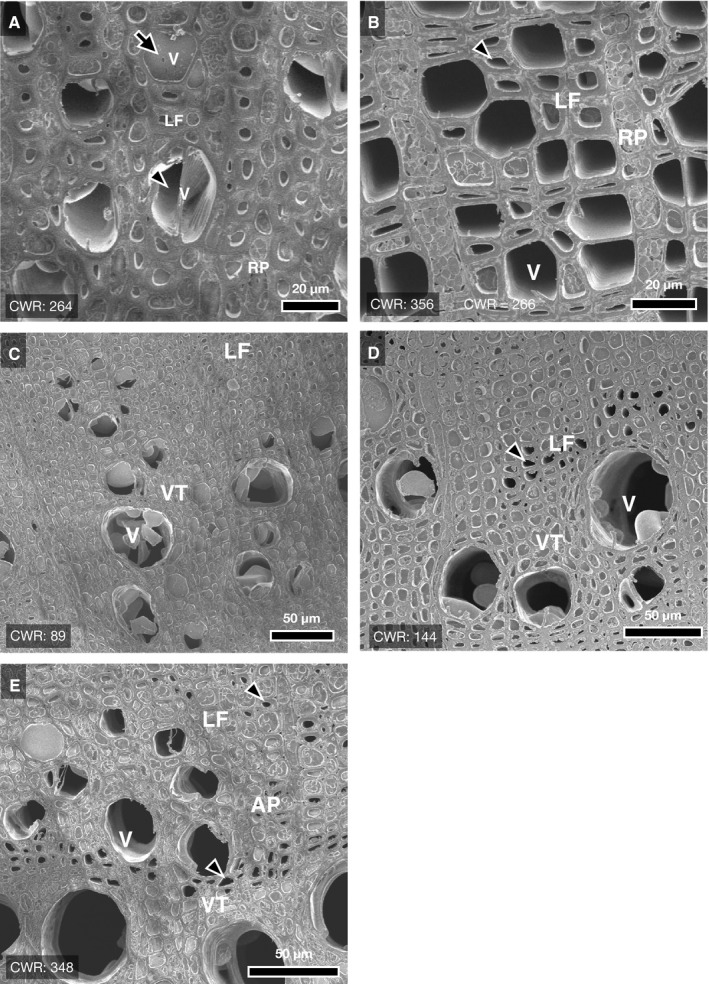
Water distribution within the xylem of *Cercidiphyllum japonicum* (A–B) and *Quercus serrata* (C–E) with the progression of bench‐drying. Estimated values of cumulative water release (CWR) are shown in each panel. In *C. japonicum*, both filled (arrow in panel A) and empty (arrowhead in panel A) vessels are under high dehydration, while all wood fibers are filled with water, and ray parenchyma cells also hold contents. Then, some wood fibers also lost water with the progress of dehydration (arrowhead, B). In *Q. serrata*, all imperforated tracheary elements (ITEs) contain water, while many vessels lost water (C). Then, under severe dehydration as almost all vessels lost water with bench‐drying (D and E), many ITEs surrounding empty vessel elements (i.e., vasicentric tracheids) contain water (D), while fiber tracheids lost their water (arrowheads in D and E). Symbols in each panels indicate cell types as follows: V, vessel; VT, vasicentric tracheid; LF, libriform fiber; AP, axial parenchyma; RP, ray parenchyma.

**Figure 8:**
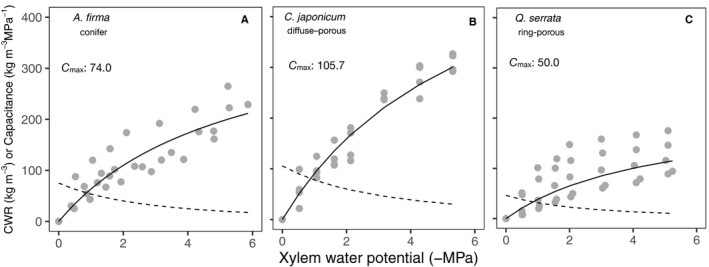
Differences in the relationships between cumulative water release (CWR), capacitance, and xylem water potential (*ψ*_x_) among (A) *Abies firma*, (B) *Cercidiphyllum japonicum*, and (C) *Quercus serrata*. The curves are obtained from whole twigs using a centrifuge. Solid lines and dashed lines indicate CWR and capacitance, respectively. The significance of the parameters of the fitted curve in each species is shown in Table 3. Values of coefficient of determination (*R* ^2^) for the CWR curves, which were calculated as 1 − Residual sum of squares / Total sum of squares with predicted values obtained using fixed effects, are 0.80, 0.96, and 0.60 in *A. firma*, *C. japonicum*, and *Q. serrata*, respectively. Maximum capacitance (*C*_max_), i.e., the capacitance at 0 MPa of water potential, is also shown in each panel.

Imperforated tracheary elements maintained water up to at least approximately 250 kg m^‐3^ and 100 kg m^‐3^ of CWR in *C. japonicum* and *Q. serrata*, respectively, indicating that the water in the lumen of ITEs was not released as capillary storage water at the early phase of water release (Figs. 6D, 6E, 7A, and 7C). Under high CWR (>300, i.e., highly dehydrated), some vessels of *C. japonicum* and ITE (wood fiber; *C. japonicum* has neither vasicentric tracheids nor fiber tracheids [Ohtani, 2000]) could hold water (Figs. 6D and 7B). Ray parenchyma cells did not lose water under such highly dehydrated conditions (Fig. 7B). Some ITEs, especially wood fiber, clearly lost water over 100 kg m^‐3^ of CWR (Figs. 7D and 8E), at which almost all vessels in *Q. serrata* lost water corresponding to µCT sequential images (Fig. 6C). With the progression of dehydration, the water in some vasicentric tracheids began to dissipate (Fig. 6E), but water was held in axial parenchyma cells (Fig. 7E).

### Water‐release curve and capacitance

We evaluated the accuracy of two methods for determining the water release curve and assumed that water release curves for whole twigs was more appropriate for estimating dehydration status in entire segments (Appendices S5–S8).

The capacitance of whole branches at full saturation of xylem (i.e., xylem water potential is nearly 0 MPa) was higher in *C. japonicum* and lower in *Q. serrata* (*C*_max_ in Fig. 8). Parameter *a* in the water release curves indicates the maximum CWR of the species. *Cercidiphyllum japonicum* had a higher parameter *a*, i.e., maximum CWR, than the other two test species, although we could not estimate the amount of water release below −6 MPa of water potential due to equipment limitation (Tables 3 and 4). No clear differences were found in parameter *b*, indicating the water potential at half of maximum CWR, between species (Tables 3 and 4).

**Table 3:** Estimated parameters of whole‐twig water release curves.

Species	Parameter	Parameter estimate	SE	*t*	df	*P*
*Abies firma*	*a*	387.1	28.2	13.7	29	<0.0001
	*b*	5.2	1.1	4.7	29	<0.0001
*Cercidiphyllum japonicum*	*a*	647.3	54.5	11.9	30	<0.0001
	*b*	6.1	0.9	6.5	30	<0.0001
*Quercus serrata*	*a*	210.5	5.4	38.7	39	<0.0001
	*b*	4.2	1.2	3.5	39	0.0011

**Table 4:** Interspecific comparison of the parameter values of the water release curves of whole twigs among the test tree species.

Parameter	Comparison	Parameter estimate	SE	*t*	df	*P*
*a*	*Abies firma* vs *Cercidiphyllum japonicum*	−272.5	49.2	−5.5	96	<0.0001
	*A. firma* vs *Quercus serrata*	183.2	29.8	4.7	96	<0.0001
	*C. japonicum* vs *Q. serrata*	455.7	42.5	10.7	96	<0.0001
*b*	*A. firma* vs *C. japonicum*	−1.1	1.6	−0.7	96	0.4862
	*A. firma* vs *Q. serrata*	1.4	1.5	1.0	96	0.3421
	*C. japonicum vs Q. serrata*	2.5	1.5	1.7	96	0.0928

Notes

Each estimated value is the difference between the estimated value of the parameter for the two species.

## DISCUSSION

### Main source of xylem water storage corresponding to dehydration status

The results of our study focused on the water behavior of the individual cell types under dehydration. Thus, it is important to note that water transferability within xylem segments should be interpreted in terms of the physical properties of the compartment of xylem associated with the anatomical structure. Water storage of fully saturated xylem with higher capacitance has been widely accepted to be determined by the water dynamics of the capillarity of matrix cells such as ITEs (Tyree and Yang, 1990; Richards et al., 2014; Pratt and Jacobsen, 2016). Our sequential CT images that followed changes in water storage in whole twigs revealed that water stored in ITEs will not be used as an initial buffer under water stress. Water stored in conduits will be utilized first. While water stored in elastic cells in xylem or inner bark is thought to alleviate further dehydration with the progress of dehydration (Tyree and Yang, 1990), the amount of parenchyma cells in xylem seemed to be too small compared to the ITEs and conduits to support the water storage (Table 1). Inability to release water from ITEs and parenchyma cells during the early phase of dehydration indicates that xylem may have less storage capacity, especially in the early phase of dehydration than has been reported. The capacitance estimated in excised xylem with psychrometers have provided useful insights about water storage in xylem (Meinzer et al., 2003; Richards et al., 2014). However, excessive evaporation from the surface of stripped samples or open conduits may induce overestimation of capacitance, which will be not correspond to the water status of xylem in situ (Fig. 8; Appendices S5–S8). Some species also do not show the quick water release of xylem in the early phase of dehydration corresponding to the capillary water storage using the centrifuge technique (Cochard et al., 2010). Further validation is needed to clarify the nature of xylem water storage.

Water storage under lower capacitance has also been believed to be determined by water dynamics of living cells, such as xylem parenchyma (Tyree and Yang, 1990; Richards et al., 2014). Shrub species distributed in regions with lower precipitation tend to have a larger fraction of axial parenchyma with denser xylem (Martinez‐Cabrera et al., 2009). Our cryo‐SEM and sequential µCT images, however, have signaled the significance of the kinetics of capillary water as the main component of water capacitance under the whole range of capacitance of each species. Jupa et al. (2016) also suggested that capillary water is a more important component of stored water under lower capacitance than elastic water that is assumed to be in xylem parenchyma cells. The fact that parenchyma cells in angiosperms retained their contents under high dehydration (Fig. 7B, E) also indicates that parenchyma cells should have a minor role as the “water server” under progressive dehydration conditions. Further investigation about the parenchyma cell behavior and mortality in relation to water shortage is needed.

The spread of cavitation from the outer xylem in *A. firma* (Fig. 4), which consists of latewood‐like cells, is consistent with several studies reporting that latewood of coniferous species has a higher vulnerability to dehydration than earlywood (Domec and Gartner, 2002; Domec et al., 2006; Dalla‐Salda et al., 2014). The maintenance of water in the wider tracheids, which should be have higher conductivity based on the Hagen–Poiseuille law, can contribute to the dehydration tolerance of coniferous species and possibly support the transpiration stream under decreasing xylem water potential.

The pattern of the water dissipation in *C. japonicum* in our study differed from that of prior work which reported that older vessels in the inner xylem were more vulnerable than newer formed vessels in the outer xylem of intact seedlings that was observed via MRI (Fukuda et al., 2015). Our experimental dehydration using cut, debarked twigs showed variation in water transferability of xylem cells without bark or the connection to the leaves, main stem, and root system. Nonetheless, it is important to note that the pathway of water transfer may differ between excised twig segments and intact plants.

### Imperforated tracheary elements as a capillary water source

Sano et al. (2011) compared the water dynamics of ITEs in living saplings of many angiosperm species by anatomical methods and found that both *C. japonicum* and *Q. crispula*, related to *Q. serrata* in this study, have conductive ITEs. Under highly dehydrated conditions, *C. japonicum* can also retain its water in the xylem within both vessels and ITEs (Fig. 7B). Thus, a functional transition concerning water transport and storage can exist between ITEs and vessels. In contrast, the species that have more complicated xylem structures, such as *Q. serrata* (Table 1), will make vasicentric tracheids specialized for storage and/or water transport under dehydrated conditions (Fig. 7C–E). These functional differences in cell types will correspond to the fine structure of pit membranes located between vessels and ITEs (Sano and Jansen, 2006; Sano et al., 2008).

Imperforated tracheary elements can possibly facilitate stabilization in tree water hydraulics. Barotto et al. (2016) reported that the higher amount of vasicentric tracheids in the xylem of *Eucalyptus* species contribute to both hydraulic conductivity and vulnerability to cavitation. Therefore, water storage in the short conduits will alleviate drastic changes in water content when water levels fluctuate, although they should have high resistance to water transferability due to segmentation of the conduits by the end walls at the connection between cells. In contrast, however, other µCT observations have revealed that ITEs barely refill, and they are not able to alleviate dehydration from vessels during dehydration in intact saplings of *Laurus nobilis* (bay laurel) or *Castanea dentata* (American chestnut) (Knipfer et al., 2017, 2019). These variations in hydraulic functional traits should be associated with fine structures of the intercellular pathway, which can determine water movement under drought. For *Q. crispula*, the pit membrane of the pit between the vessel element and vasicentric tracheid may have an impermeable structure due to the accumulation of microfibrils (Sano et al., 2008). Thus, on the assumption that the pit structure in *Q. serrata* is similar to that in *Q. crispula*, the stored water remaining in the xylem of *Q. serrata* under very lower xylem water potential (Fig. 7) might be unavailable for plant survival because of the low transferability of water in the ITEs. Further direct observations of water dynamics will provide new insights about the hydraulic roles of ITEs.

Compared to the case in broadleaved species, the manner of water dissipation in *A. firma* (coniferous species) seems to be inconsistent with its predicted hydraulic tolerance from cell dimensions; that is, wider conduits are more vulnerable than narrower conduits to dehydration (Hacke et al., 2001). Moreover, tracheids in earlywood generally have more inter‐tracheid connections via bordered pits than in latewood (Ohtani, 2000; Richter et al., 2004), which could induce water depletion from earlywood. However, as mentioned previously, some studies reported that latewood is more vulnerable than earlywood in coniferous species (Domec and Gartner, 2002; Domec et al., 2006; Dalla‐Salda et al., 2014). The differences in the structure and kinetics of the pit membrane in bordered pits between earlywood and latewood can explain this water behavior of coniferous xylem under progression to dehydration (Domec et al., 2006; Delzon et al., 2010). The flexible bordered pit membrane of tracheids in earlywood may play a role as a “valve” to preserve water in the main conduits (Yazaki et al., 2017).

### Water dynamics of stored water corresponding to individual water use

For an individual tree, higher capacitance at operating water potential (the range of the water potential of the species in native environmental condition) in xylem contributes to the maintenance of daytime water potential at high levels (Meinzer et al., 2009; Richards et al., 2014). Our results revealed the main water source of capacitance in the early dehydration phase is water in vessels (Figs. 4, 5, 6). Thus, more vessels will contribute to higher capacitance when comparing the anatomical structure of *C. japonicum* with that of *Q. serrata* (Table 1). In other words, the large amount of water needed to buffer the changes in water potential during early daytime can be released primarily from vessels (Fig. 6; Appendix S8). Alternatively, xylem with higher capacitance has lower drought tolerance in hydraulic function due to the sparse xylem (Hacke et al., 2001; Jacobsen et al., 2005; Meinzer et al., 2009). In addition, species with high capacitance tend to have a lower water potential margin (the differences between daily minimum water potential and water potential that induce hydraulic dysfunction) (Meinzer et al., 2009; Johnson et al., 2012). Our results can explain that the depletion of stored water will directly induce xylem hydraulic dysfunction involving the reduction of water potential of leaves.

Generally, the species with denser xylem tend to have higher drought resistance, owing to the dehydration delay in narrower conduits or high rigidity of the cell wall under negative pressure (Hacke et al., 2001; Jacobsen et al., 2005; Chave et al., 2009). Our results suggest that ITEs, which are the main determinant of wood density (Fortunel et al., 2014), will support the functional tolerance of xylem conduits, especially under prolonged drought. The daily minimum water potentials in the leaves of adult tree of *C. japonicum* and *Q. serrata* are approximately –1.2 MPa and –2.0 MPa, respectively (Saito et al., 2003; Fukuda et al., 2015). Our results indicate the possibility of daily hydraulic dysfunction of vessels in the field (Fig. 6; Appendix S8), although vessels have been thought to be less vulnerable than expected from destructive measurements (Cochard et al., 2015). The diminishment of the transpiration stream caused by dysfunction of wide vessels under high dehydration may be eased by ITEs. Direct observations of the seasonal changes in water distribution in ITEs in xylem are scarce, but ITEs have been reported in newly formed xylem contain water during the growing period (Utsumi et al., 1996, 1998). Such water in ITEs can be expected to facilitate water transport of vessels (Pratt et al., 2015). Comprehensive study of the direct observation of water dynamics along various wood structures corresponding to the hydraulic network of cells in living trees will clarify the hydraulic function of the wood structure. The phylogenetic exploration of wood structure associated with water storage could lead to better predictions of tree mortality.

## CONCLUSIONS

The combination of cryo‐SEM and sequential µCT observations of water in xylem indicated that the anatomical structure of xylem should largely determine the transferability of water that help support the transpiration stream during the progression of drought, while the intrinsic property of wood density provides xylem dehydration tolerance. The direct linkage between capacitance and water dynamics in xylem tissues during dehydration leads to the expectation that water in conduits should determine the water storage in the early phase of dehydration, while water in ITEs should play an important role in the water storage of xylem under prolonged dehydration. Although the transferability of water in the xylem of twigs shown in this study may be dissimilar to that in a living branch, which has bark and a transpiration stream, our results indicate that the differential hydraulic vulnerability of conduits would partly determine the mode of water storage of the species. With respect to the estimation of xylem capacitance, our results demonstrated that the storage role of xylem in the early phase of dehydration should be reconsidered. Our results suggest that species that can store transferable water in xylem conduits have an advantage in supporting transpiration from water stress when water availability fluctuates, which may become more likely with climate change.

## AUTHOR CONTRIBUTIONS

Y.K. designed experiments, conducted experiments, and analyzed data and wrote the manuscript. D.L. discussed data presentation and wrote the manuscript. T.A. and W.M. examined µCT images. K.D. statistical analyzed data. M.N. and S.S.‐T. measured capacitance. T.H. [Taneda] measured vessel length. O.M. measured cavitated area. OMY, MN, T.H. [Taneda], T.H. [Tobita], and F.K. designed experiments.

## Supporting information


**APPENDIX S1.** Representative images of the transverse section of (A) *Cercidiphyllum japonicum* and (B) *Quercus serrata*.

**APPENDIX S2.** Typical sequential µCT images showing the distribution of water in the xylem of *Cercidiphyllum japonicum* (diffuse‐porous) and the water status of xylem of short segments (4 cm long) that have been bench‐dried.

**APPENDIX S3.** Typical sequential µCT images showing the distribution of water in the xylem of *Quercus serrata* (ring‐porous) and the water status of xylem of short segment (4 cm long) that have been bench‐dried.

**APPENDIX S4.** Comparison between µCT and cryo‐SEM images.

**APPENDIX S5.** Comparison of water‐release curves and capacitances between centrifugal and psychrometrical methods (text).

**APPENDIX S6.** Comparison of water‐release curves and capacitances between centrifugal and psychrometrical methods.

**APPENDIX S7.** Estimated parameters for water release curves for a whole twig or small segments.

**APPENDIX S8.** Comparison of estimated value of cumulated water release (CWR) at given water potentials for each species between two water release curves.
