# Supplementary material for: Imperforate tracheary elements and vessels alleviate xylem tension under severe dehydration: insights from water release curves for excised twigs of three tree species
Source: Am J Bot. 2020 Aug 11;107(8):1122–35. doi: 10.1002/ajb2.1518 (PMC7496847; doi:10.1002/ajb2.1518)

APPENDIX S1

Representative images of transverse section of (A) *Cercidiphyllum japonicum* and (B) *Quercus serrata*. Arrowheads in panel B indicate typical axial parenchyma cells. V, vessel; LF, libriform fiber; VT, vacisentric tracheid; AP, axial parenchyma; RP, ray parenchyma.


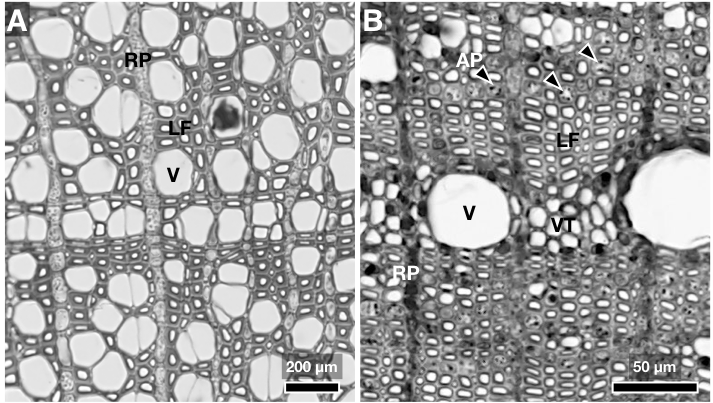

Supplement: Supplementary file 1 — APPENDIX S1. Representative images of the transverse section of (A) Cercidiphyllum japonicum and (B) Quercus serrata. [file AJB2-107-1122-s001.docx]
