# Supplementary material for: Imperforate tracheary elements and vessels alleviate xylem tension under severe dehydration: insights from water release curves for excised twigs of three tree species
Source: Am J Bot. 2020 Aug 11;107(8):1122–35. doi: 10.1002/ajb2.1518 (PMC7496847; doi:10.1002/ajb2.1518)
Supplement: Supplementary file 2 — APPENDIX S2. Typical sequential µCT images showing the distribution of water in the xylem of Cercidiphyllum japonicum (diffuse‐porous) and the water status of xylem of short segments (4 cm long) that have been bench‐dried. [file AJB2-107-1122-s002.docx]

APPENDIX S2

Typical sequential µCT images showing the distribution of water in the xylem of *Cecidiphyllum japonicum* (diffuse-porous) and the water status of xylem of short segments (4 cm long) that have been bench-dried. Estimated values of CWR are also shown in each panel. New occurrences of cavitation are shown in panel G (from panel D to E) and H (from panel E to F). The manner of water dissipation is almost similar to those of longer segments shown in the main text. Scale bar: 200 µm.
