# Supplementary material for: Imperforate tracheary elements and vessels alleviate xylem tension under severe dehydration: insights from water release curves for excised twigs of three tree species
Source: Am J Bot. 2020 Aug 11;107(8):1122–35. doi: 10.1002/ajb2.1518 (PMC7496847; doi:10.1002/ajb2.1518)
Supplement: Supplementary file 3 — APPENDIX S3. Typical sequential µCT images showing the distribution of water in the xylem of Quercus serrata (ring‐porous) and the water status of xylem of short segment (4 cm long) that have been bench‐dried. [file AJB2-107-1122-s003.docx]

APPENDIX S3

Typical sequential µCT images showing the distribution of water in the xylem of *Quercus serrata* (ring-porous) and the water status of xylem in short segments (4 cm long) that have been bench-dried. Typical sequential images of one sample (A–D) and another (E–F) are shown. Note that cavitation is shown in the wood fiber (the area within the white-dotted line) in panel F, as opposed to panel E. The manner of water dissipation is almost similar to those of longer segments shown in the main text. Scale bar: 200 µm.
