# Supplementary material for: Imperforate tracheary elements and vessels alleviate xylem tension under severe dehydration: insights from water release curves for excised twigs of three tree species
Source: Am J Bot. 2020 Aug 11;107(8):1122–35. doi: 10.1002/ajb2.1518 (PMC7496847; doi:10.1002/ajb2.1518)
Supplement: Supplementary file 4 — APPENDIX S4. Comparison between µCT and cryo‐SEM images. [file AJB2-107-1122-s004.docx]

APPENDIX S4

Comparison between µCT (A or C) and cryo-SEM images (B or D). Both images were taken from the same twigs of *Quercus serrata* (A and B) or *Abies firma* (C and D). Letters in panels A and B indicate identical location marked by the arrow and arrowheads. The arrowheads and arrows indicate similar and different water content levels, respectively. The cryo-SEM images of *Q. serrata* and *A. firma* frozen just after scanning of µCT revealed many similarities of water distribution in its conduits to the µCT images (A and B, or C and D). However, there were some notable differences (see arrowhead) in the appearance of water between the images derived from two methods, especially for *Q. serrata*. Some water-filled vessels observed with µCT appeared dissipated in the picture of cryo-SEM (e.g., the arrows of C and D), while some cavitated vessels observed with µCT seemed to be filled with water in the cryo-SEM picture (e.g., the arrow of E).
