# Supplementary material for: Imperforate tracheary elements and vessels alleviate xylem tension under severe dehydration: insights from water release curves for excised twigs of three tree species
Source: Am J Bot. 2020 Aug 11;107(8):1122–35. doi: 10.1002/ajb2.1518 (PMC7496847; doi:10.1002/ajb2.1518)
Supplement: Supplementary file 5 — APPENDIX S5. Comparison of water‐release curves and capacitances between centrifugal and psychrometrical methods (text). [file AJB2-107-1122-s005.docx]

Appendix S5

Comparison of water release curves and capacitances between centrifugal and psychrometrical methods

The water release curves and the corresponding parameters of water release curves determined in whole twig or small segments for each of the three test species are shown in Appendices S6–8. Overall, the initial slope of the water release curve was steeper in small segments using the psycrometer method than in whole twigs using the centrifuge method, indicating the amount of released water at the range of low water potential was higher in small segments than in whole twigs (Appendix S6). The cumulative water release (CWR) in whole twigs tended to increase with a decrease in water potential within -6 MPa (i.e., increase in tension along xylem), especially for *Abies firma* and *Cercidiphyllum japonicum* (Appendix S6A and B). In contrast, the water release curve for small segments seemed to be asymptotic within the given water potential. Parameter *b* in the water release curve is thought to indicate the water potential at which the source of water release changes from capillary storage only (Phase I) to capillary storage with elastic storage and cavitation release (Phase II) (Jupa et al., 2016). Parameter *b* in the small segments is lower than in whole twigs of all studied species (Appendix S7), indicating early transition of water release phase with dehydration.

We also evaluated the CWR values in the curve using the two methods corresponding to two conditions of water release, i.e., (1) within Phase I at −0.5 MPa of water potential, (2) at the water potential during the day (Appendix S8). Each of the estimated values of CWR is higher in small segments than in whole twigs of each species. The water distribution in xylem corresponding to each estimated CWR seemed to be consistent with the value from whole twigs because too many conduits should be empty during the day when water in the xylem is released in accordance with the water release curve in small segments (e.g., almost all vessels may be empty during daytime in *Quercus. serrata*; Fig. 6C in the main text). Thus, we assumed that water release curves for whole twigs are more appropriate to estimate the dehydration status in entire segments.

**Literature Cited**

Jupa, R., L. Plavcová, V. Gloser, and S. Jansen. 2016. Linking xylem water storage with anatomical parameters in five temperate tree species. *Tree Physiology* 36: 756–69.
