# Supplementary material for: Imperforate tracheary elements and vessels alleviate xylem tension under severe dehydration: insights from water release curves for excised twigs of three tree species
Source: Am J Bot. 2020 Aug 11;107(8):1122–35. doi: 10.1002/ajb2.1518 (PMC7496847; doi:10.1002/ajb2.1518)
Supplement: Supplementary file 6 — APPENDIX S6. Comparison of water‐release curves and capacitances between centrifugal and psychrometrical methods. [file AJB2-107-1122-s006.docx]

APPENDIX S6

Comparison of water release curves and capacitances between centrifugal and psychrometrical methods. The relationships between cumulative water release (CWR), capacitance, and xylem water potential (*ψ*_x)_ among (A, D) *Abies firma*, (B, E) *Cercidiphyllum japonicum*, and (C, F) *Quercus serrata* are shown. The curves in panels A–C were obtained from whole twigs using a centrifuge (i.e., similar to the figures in the main text) and those in panels D–F were from the small pieces of samples using a psychrometer. Solid lines and dashed lines indicate CWR and capacitance, respectively. Maximum capacitance (*C*_max_), i.e., the capacitance at 0 MPa of water potential is also shown in each panel.
