# Supplementary material for: Imperforate tracheary elements and vessels alleviate xylem tension under severe dehydration: insights from water release curves for excised twigs of three tree species
Source: Am J Bot. 2020 Aug 11;107(8):1122–35. doi: 10.1002/ajb2.1518 (PMC7496847; doi:10.1002/ajb2.1518)
Supplement: Supplementary file 7 — APPENDIX S7. Estimated parameters for water release curves for a whole twig or small segments. [file AJB2-107-1122-s007.docx]

APPENDIX S7. Estimated values for parameters of water release curves for a whole twig or small segments.

| Species | Method | Parameter | Parameter estimate | SE | *t* | df | *P* |  |
| --- | --- | --- | --- | --- | --- | --- | --- | --- |
| *Abies firma* | Twig (Centrifuge) | *a* | 387.1 | 28.2 | 13.7 | 29 | <0.0001 | |
|  |  | *b* | 5.2 | 1.1 | 4.7 | 29 | <0.0001 | |
|  | Segments (Psychrometer) | *a* | 474.6 | 25.3 | 18.7 | 24 | <0.0001 | |
|  |  | *b* | 1.9 | 0.6 | 3.1 | 24 | 0.0051 | |
|  |  |  |  |  |  |  |  | |
| *Cercidiphyllum japonicum* | Twig (Centrifuge) | *a* | 647.3 | 54.5 | 11.9 | 30 | <0.0001 | |
|  |  | *b* | 6.1 | 0.9 | 6.5 | 30 | <0.0001 | |
|  | Segments (Psychrometer) | *a* | 263.9 | 30.2 | 8.7 | 15 | <0.0001 | |
|  |  | *b* | 0.7 | 0.1 | 7.8 | 15 | <0.0001 | |
|  |  |  |  |  |  |  |  | |
| *Quercus serrata* | Twig (Centrifuge) | *a* | 210.5 | 5.4 | 38.7 | 39 | <0.0001 | |
|  |  | *b* | 4.2 | 1.2 | 3.5 | 39 | 0.0011 | |
|  | Segments (Psychrometer) | *a* | 307.5 | 21.3 | 14.4 | 13 | <0.0001 | |
|  |  | *b* | 3.0 | 0.4 | 7.1 | 13 | <0.0001 | |
