# Supplementary material for: Imperforate tracheary elements and vessels alleviate xylem tension under severe dehydration: insights from water release curves for excised twigs of three tree species
Source: Am J Bot. 2020 Aug 11;107(8):1122–35. doi: 10.1002/ajb2.1518 (PMC7496847; doi:10.1002/ajb2.1518)
Supplement: Supplementary file 8 — APPENDIX S8. Comparison of estimated value of cumulated water release (CWR) at given water potentials for each species between two water release curves. [file AJB2-107-1122-s008.docx]

APPENDIX S8. Comparison of the estimated cumulated water release (CWR) at given water potentials for each species based two water release curves.

|  |  | CWR (kg m^-3^) | |
| --- | --- | --- | --- |
| Species | Method | Phase I -0.5 MPa | Daytime |
| *Abies firma* | Whole twigs (Centrifuge) | 33.8 | 86.2 |
|  | Small segments (Psychrometer) | 98.2 | 208.4 |
|  |  |  |  |
| *Cercidiphyllum japonicum* | Whole twigs (Centrifuge) | 48.9 | 106.1 |
|  | Small segments (Psychrometer) | 88.7 | 149.7 |
|  |  |  |  |
| *Quercus serrata* | Whole twigs (Centrifuge) | 22.3 | 67.8 |
|  | Small segments (Psychrometer) | 43.9 | 122.9 |

*Notes*: Each value for daytime water potential is based on literatures values: −1.5 MPa in *A. firma* (Taneda and Tateno, 2005), −1.2 MPa in *C. japonicum* (Fukuda et al., 2015), −2.0 MPa in *Q. serrata* (Saito et al., 2003).
